# Macular choroidal thickness, volume, and vascularity index in patients with systemic sclerosis

**DOI:** 10.1007/s00417-023-06342-4

**Published:** 2023-12-22

**Authors:** Barbara Pieklarz, Ewa Gińdzieńska-Sieśkiewicz, Izabela Zawadzka, Magdalena Bagrowska, Joanna Daniluk, Marcin Palewski, Agnieszka Zonenberg, Otylia Kowal-Bielecka, Joanna Konopińska, Diana Anna Dmuchowska

**Affiliations:** 1 Ophthalmology Department, Medical University of Bialystok, 24a M.Sklodowskiej-Curie, 15-276 Bialystok, Poland; 2 Department of Rheumatology and Internal Diseases, Medical University of Bialystok, 24a M. Sklodowskiej-Curie, 15-276 Bialystok, Poland

**Keywords:** Choroidal vascularity index, Choroidal thickness, Systemic sclerosis, Choroidal microcirculation

## Abstract

**Purpose:**

The aim of this study was to investigate choroidal parameters in patients with systemic sclerosis (SSc) using enhanced depth imaging spectral-domain optical coherence tomography (EDI-SD-OCT) and to determine their relationships with clinical variables and ocular features.

**Methods:**

Thirty-three patients with SSc and 40 controls were enrolled. The groups did not differ with regard to age, sex, and axial length. The mean choroidal thickness and volume were obtained in each conventional Early Treatment of Diabetic Retinopathy Study grid subfield. The choroidal vascularity index (CVI), which provides a quantitative analysis of vasculature by calculating the proportion of the luminal area (LA) to the total choroidal area (TCA), was determined.

**Results:**

Lower choroidal thickness and volume were observed in the SSc group. The CVI was significantly higher in SSc patients, whereas the TCA, LA, and stromal area were significantly lower in the SSc group; however, the significant difference of the stromal component was more pronounced than that of the luminal component. Regression analyses did not identify any clinical factors associated with the CVI (except Ca-blocker use), central macular thickness, or volume. No significant differences in choroidal parameters were found within the SSc subtypes (diffuse cutaneous systemic sclerosis (dcSSc) vs. limited cutaneous systemic sclerosis (lcSSc)), or between eyes stratified according to SSc pattern (early, active, or late) using nailfold capillaroscopy (*p* > 0.05 for all).

**Conclusion:**

Our results, with notably higher CVI values, may shed new light on choroidal impairment in patients with SSc. Stromal involvement appeared to dominate the vascular component.

**Supplementary Information:**

The online version contains supplementary material available at 10.1007/s00417-023-06342-4.

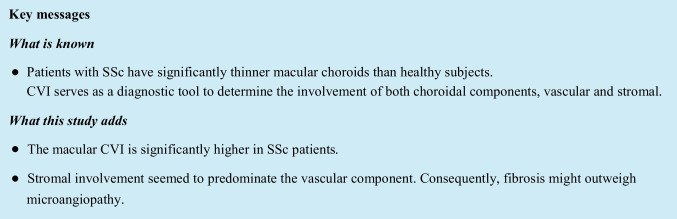



## Introduction

Systemic sclerosis (SSc) is a complex, progressive, autoimmune connective tissue disease with partially understood triggers. The pathomechanisms first involve microvascular damage, followed by autoimmune response, inflammation, and diffuse fibrosis [1]. SSc can be generally classified based on the extent of skin involvement. Cases with proximal skin involvement are classified as diffuse cutaneous systemic sclerosis (dcSSc), whereas those with skin involvement affecting the limbs distal to the elbows and knees, with or without neck and face involvement, are classified as limited cutaneous systemic sclerosis (lcSSc) [2]. Both dcSSc and lcSSc patients are at risk of internal organ involvement [3].

Many ocular manifestations involving anterior and posterior segments have been reported in SSc patients [4–6], and there is no doubt that retinal and choroidal microcirculation impairments occur in SSc patients [7–10]. It has been hypothesized that generalized vasculopathy causes alterations in the posterior segment and uveal tract, whereas fibrosis-related impairment is more likely to affect the anterior segment and adjacent area [6].

The choroid is a highly vascularized tissue that provides 85% of the total ocular blood flow [11]. Choroidal circulation is characterized by a high level of blood flow with low oxygen extraction in contrast to retinal circulation [11]. The end-arterial nature of the choroidal vasculature makes this layer vulnerable to inflammation and ischemia in multisystemic diseases [12]. Therefore, it seems that the choroidal vasculature is ideal for the observation of generalized arteriolar and capillary injury in SSc [13]. Enhanced depth imaging optical coherence tomography (EDI-OCT) is a noninvasive, rapid, objective, and reliable diagnostic modality for imaging choroidal alterations [14, 15]. In recent years, choroidal thickness (CT) has been proposed as an inflammatory biomarker in systemic autoimmune diseases, especially those with vascular components [16]. Most studies on choroidal thickness have found that patients with SSc have a significantly thinner macular choroid than healthy subjects, presumably as a result of chronic vascular damage [7–9, 17–20]. Choroidal thickness is a rough estimate rather than an accurate marker of choroidal status and is dependent on various physiological and pathological factors, including age, ethnicity, sex, refraction, and axial length [21]. Hence, we determined not only the choroidal thickness but also the choroidal vascularity index (CVI), which is a novel OCT-based choroidal quantitative parameter that provides more detailed information about the vascular component of the choroid across all layers: the choriocapillaris, Sattler’s layer, and Haller’s layer. The CVI provides a quantitative analysis of the vasculature by calculating the proportion of the luminal area to the total choroidal area. The current literature suggests that the CVI has less variability and is influenced by fewer physiological factors than choroidal thickness. Therefore, it can be considered a relatively stable parameter for the evaluation of changes in the choroidal vasculature [22–24]. It has been proposed as a potential biomarker for establishing early diagnosis, monitoring disease progression, and prognosticating patients [23–25]. As other authors have emphasized, the CVI should not be viewed as an isolated marker, but as an addition to existing parameters such as CT [25]. Therefore, we considered not only the choroidal thickness but also the choroidal volume, in order to take into account irregularities in the choroidal–scleral junction [26].

In the current study, we aimed to investigate choroidal parameters and to determine their relationships with clinical variables and ocular features. We hypothesized that patients with SSc might demonstrate alterations in choroidal morphology. Moreover, assessment of the CVI may help to differentiate whether vascular, stromal, or both components are involved in the pathogenesis of choroidal changes. This is the first ever study to address this pathogenic issue.

## Material and methods

This prospective, single-center cross-sectional study was conducted between March 2021 and March 2022 at the Ophthalmology Department of the Medical University of Bialystok. The study involved 33 adult SSc patients (66 eyes) admitted to the Department of Rheumatology and Internal Diseases of the Medical University of Bialystok. Diagnoses were made according to the 2013 ACR/EULAR SSc criteria [27], and subtypes were ascertained as diffuse or limited. The protocol of the study was approved by the local Bioethics Committee at the Medical University of Bialystok (decision no APK.002.109.2021), and the study was conducted in accordance with the Declaration of Helsinki. Informed written consent was obtained from each patient.

The control group was composed of 80 eyes from 40 patients undergoing routine ophthalmological assessments. SSc patients and controls did not differ with regard to age, sex, and axial length (AL). The following detailed ophthalmologic examination, including refraction, best corrected visual acuity (BCVA) in Snellen converted to log MAR, intraocular pressure (IOP) measured with a Pascal dynamic contour tonometer (DCT; Zeimer Ophthalmic Systems AG, Port, Switzerland), slit-lamp biomicroscopy, AL measured with a Tomey OA-2000 biometer (Nagoya, Japan), dilated fundus examination, and enhanced depth imaging spectral-domain optical coherence tomography (EDI SD-OCT, Heidelberg Engineering GmbH, Heidelberg, Germany; 2016), was performed on all participants. Blood pressure was measured in the sitting position after 5 min of rest, and OCT images were obtained immediately afterwards for all patients.

Data regarding age, sex, disease duration, autoantibody profile, current smoking status, and details of systemic treatment were recorded. History of digital ulcers (present or past), the presence of interstitial lung disease (ILD) confirmed by high-resolution computed tomography (HRCT) of the lungs, cardiac involvement (elevated N-terminal pro b-type natriuretic peptide (NT-proBNP or heart fibrosis upon magnetic resonance imaging (MRI)), and joint involvement (arthalgia or joint swelling) were also included in the analysis. Nailfold capillaroscopy (NFC) was performed using a CapillaryScope 200 Dino-lite Digital microscope (MEDL4N PRO capillaroscopy equipment) and stratified based on capillaroscopic characteristics (capillary density, capillary dimension, abnormal capillary morphology, and presence or absence of hemorrhages) as “early,” “active,” or “late” SSc pattern, as proposed by Cutolo et al. [28]. Blood parameters, including C-reactive protein (CRP) and erythrocyte sedimentation rate (ESR; after 2 h), were measured.

Exclusion criteria encompassed the presence of fundus pathology, ametropia ≥ 3 diopters, phacoemulsification less than 12 months prior to examination, history of posterior segment surgery, retinal laser treatment, and poor quality of OCT scans (< 25 dB). To exclude preexisting fundus abnormalities, fundoscopy in mydriasis was performed and color fundus photographs were obtained and analyzed for each patient. Additionally, each OCT scan was carefully evaluated with emphasis on Bruchs membrane/RPE and choroidal abnormalities to detect any features of the pachychoroid, including pachychoroid pigment epitheliopathy [29].

### OCT image acquisition and analysis

**Fig. 1:**
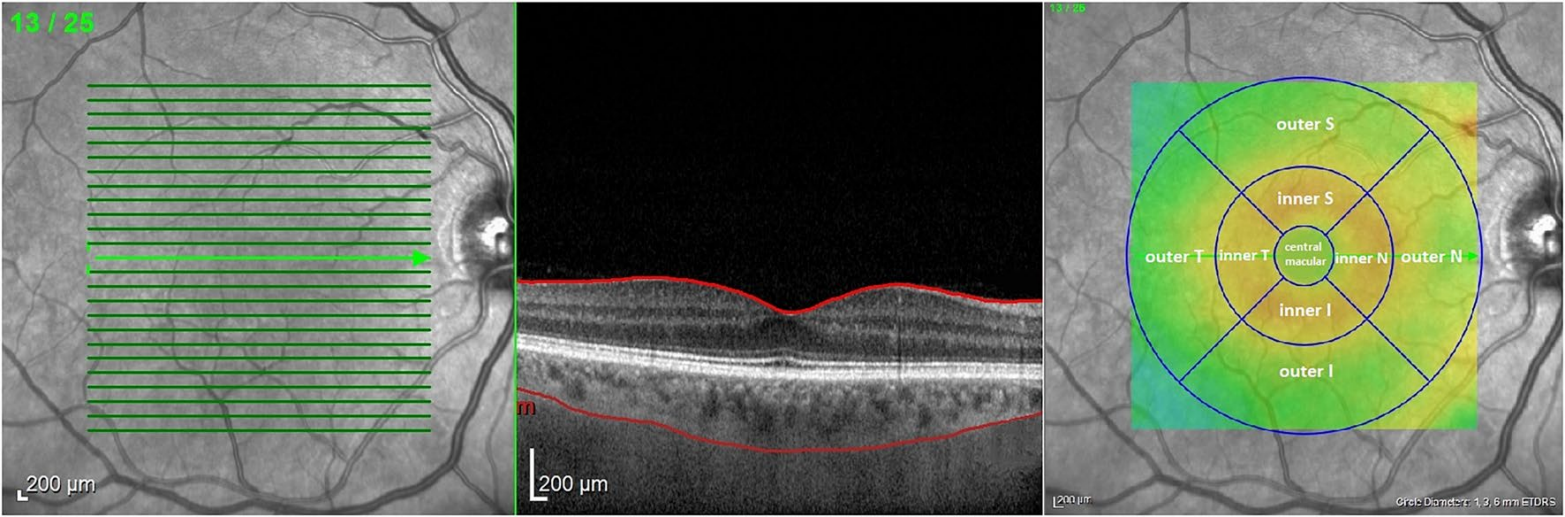
Examined choroidal and retinal area overlaid on fundus image

OCT images were captured in mydriasis during the same time interval (12 pm–3 pm) to avoid diurnal variation in choroidal parameters. The EDI-SD-OCT imaging protocol was composed of 25 raster scans (20° × 20°) and a linear 30° B-scan centered at the fovea. Choroidal thickness and volume were determined in the same manner as in our previous study [23]. Briefly, the internal limiting membrane (ILM) and Bruch’s membrane (BM) were detected automatically, while the choroidal–scleral junction (CSJ) was manually marked on each scan by the same experienced examiner (MP). Retinal parameters were calculated from the ILM to the BM and choroidal parameters from the BM to the CSJ. Average thickness and volume maps were created automatically according to the conventional ETDRS grid with nine subfields: central macular subfield (central field within a 500-µm radius), four inner subfields (within a 500–1500-µm radius), and four outer subfields (within a 1500–3000-µm radius) (Fig. 1) [30]. Separate maps were created for retinal thickness and volume, as well as for the sum of the retinal and choroidal thickness and volume. Values of the choroidal parameters were calculated by subtracting retinal parameters from the summed retinal and choroidal parameters. SFCT was defined as the distance between the BM and the CSJ at the fovea and was measured automatically.

**Fig. 2:**
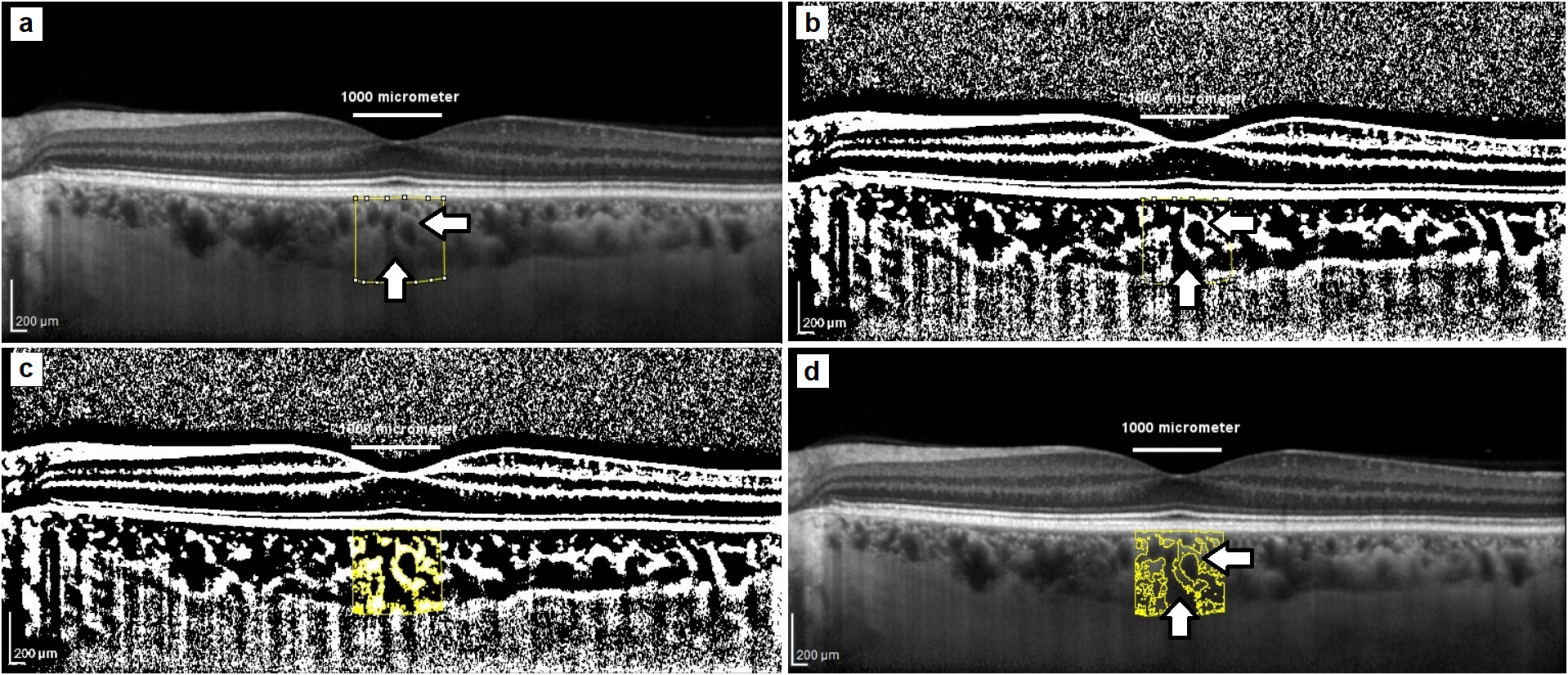
Image binarization of choroid: luminal area (vertical arrows) and stromal area (horizontal arrows). **a** Total choroidal area marked on original enhanced depth imaging (EDI) SD-OCT scan. Measurement area was defined as 1000 μm in width. **b** Niblack auto local threshold tool applied. **c** Highlighted luminal area using color threshold tool. **d** Overlay of luminal area on original OCT scan

Binarization of the choroidal area (Fig. 2) was performed by two of the authors (BP and AZ). The macular region was scanned using a single horizontal line scan (30°) centered on the fovea, with 100 frames averaged in a B-scan. Only high-quality scans (defined as scans with a signal strength of more than 25 dB (ranging from 0 = poor to 40 = excellent)) were used for analysis. Images were analyzed with ImageJ public domain software (https://imagej.nih.gov/ij/) using the protocol previously described by Sonoda et al. and Agrawal et al. [31, 32]. with a few modifications. The most important modification concerned the setting of the scale, which considered the stretching of images to avoid erroneous quantification of the measured area [33]. Damian et al. analyzed nonstretched OCT scans (1 × 1 µm) to overcome the erroneous quantification of choroidal parameters [34]. However, an image presented in a 1:1 pixel aspect ratio (stretched axially) has better detailed visualization of a structure than a 1 × 1 µm image (OCT sampling density is higher in the axial direction than the transverse) [33]. Therefore, the scale was set considering pixel aspect ratio to reflect the actual size of the measured area. A detailed step-by-step image analysis algorithm is provided in the Supplementary Material. The measurement area was defined as 1000 μm in width and centered on the fovea. The total choroidal area (TCA) was selected from the outer boundary of the RPE–BM layer to the choroidal–scleral junction using the polygon selection tool. The images were converted to 8-bit images to allow the application of the Niblack auto local threshold tool. The binarized images were reconverted to RGB images to allow the color threshold tool to be used to select dark pixels that represented vascularized areas. The luminal area (LA) and TCA were measured, while the stromal area (SA) was calculated by subtracting LA from TCA. The CVI was determined as the ratio of LA to TCA (%). The interobserver reproducibility of the measurements was assessed by measuring the intraclass correlation coefficient (ICC) and absolute agreement. The ICC values for the CVI, TCA, and LA measurements were > 0.87.

### Statistical analysis

Analyses were performed using R 4.0.5. statistical software (R Core Team (2021). R: Language and environment for statistical computing by R Foundation for Statistical Computing, Vienna, Austria). Data are presented as n (%) for nominal variables and as means ± SD or medians (Q1; Q3) for continuous variables, depending on normality of distribution (validated via Shapiro–Wilk test and based on skewness and kurtosis values). Comparisons among groups were made using chi-squared tests or Fisher’s exact tests for nominal data, as well as t-tests, ANOVA, or Kruskal–Wallis tests for continuous variables, as appropriate. Post hoc comparisons were based on Tukey tests. For the comparison of choroidal parameters between groups, Benjamini–Hochberg correction for multiple comparisons was applied. Additionally, linear regression analysis was performed to verify the associations between the CVI, central macular choroidal thickness, and volume, as well as demographic, clinical, and ocular features. All calculations were based on *α* = 0.05.

## Results

A total of 66 eyes of 33 SSc patients and 80 eyes of 40 healthy control subjects were enrolled in this study. The groups did not differ with regard to age, sex, AL, smoking status, or visual acuity. However, differences in mean arterial pressure (MAP) and IOP were found. A total of 22 (66.66%) patients presented dcSSc, and 11 (33.33%) had lcSSc. There were no significant differences between the two subtypes in terms of age, sex distribution, AL, smoking status, or visual acuity, but significant differences were found in MAP and IOP. Detailed demographic and clinical data are shown in Table 1 (control group vs. SSc group) and Table 2 (control group vs. dcSSc vs. lcSSc).

**Table 1:** Demographic and clinical characteristics of SSc patients and control group

Variable	SSc group	Control group	*p*
Number of patients	33	40	
Number of eyes	66	80	
Age, years (mean ± SD)	50.97 ± 12.27	50.43 ± 10.52	0.841^2^
Sex, F, *n* (%)	24 (72.7)	22 (55.0)	0.188
Sex, M, *n* (%)	9 (27.3)	18 (45.0)	
MAP, mean ± SD	86.47 ± 9.24	97.21 ± 12.43	** < 0.001**^**2**^
Nicotine, *n* (%)	3 (9.1)	6 (15.0)	0.484^1^
logMAR, median (Q1;Q3)	0.00 (0.00; 0.00)	0.00 (0.00; 0.00)	0.686^3^
IOP (mmHg), mean ± SD	13.94 ± 3.22	15.37 ± 2.13	**0.007**^**2**^
AL (mm), mean ± SD	23.15 ± 0.82	23.39 ± 0.97	0.104^2^
Duration of the disease (years), median (Q1;Q3)	4.00 (2.00; 10.00)		
Pulmonary involvement, *n* (%)	22 (66.7)		
Cardiac involvement, *n* (%)	11 (33.3)		
Joint involvement, *n* (%)	16 (48.5)		
Digital ulcers (present/history), *n* (%)	11 (33.3)		
CRP (mg/l), median	1.45 (1.00; 3.43)		
ESR (mm/2 h), median	27.00 (18.00; 39.00)		
Anti-Scl70 positive, *n* (%)	16 (53.3)		
Anti-centromere positive, *n* (%)	7 (23.3)		
Other Abs positive, *n* (%)	13 (43.3)		
NFC (active/early/late; number of eyes)	30/18/18		

Abbreviations: *Abs* antibodies, *AL* axial length, *CRP* C-reactive protein, *ESR* erythrocyte sedimentation rate, *F* female, *h* hours, *IOP* intraocular pressure, *M* male, *MAP* mean arterial pressure, *NFC* nailfold capillaroscopy, *SSc* systemic sclerosis Significant difference were tested using chi-square test or Fisher exact test^1^ for nominal variables and with *t*-test^2^ or Mann–Whitney *U* test^3^ for continuous variables, *p* < 0.05 was considered statistically significant (highlighted with bold)

**Table 2:** Demographic and clinical characteristics of control group and SSc subtypes

Variable	Control group	dSSc	lSSc	*p*	Post hoc		
					Control vs. dSSc	Control vs. ISSc	dSSc vs. ISSc
Number of patients	40	22	11				
Number of eyes	80	44	22				
Age, years (mean ± SD)	50.43 ± 10.52	51.41 ± 13.92	50.09 ± 8.55	0.933^2^			
Sex, F, *n* (%)	22 (55.0)	14 (63.6)	10 (90.9)	0.091^1^			
Sex, M, *n* (%)	18 (45.0)	8 (36.4)	1 (9.1)				
MAP, mean ± SD	97.21 ± 12.43	87.63 ± 10.44	84.13 ± 6.01	0.001^2^	**0.009**	**0.005**	0.694
Nicotine, *n* (%)	6 (15.0)	2 (9.1)	1 (9.1)	0.885^1^			
logMAR, median	0.00	0.00	0.00	0.919^3^			
IOP (mmHg), mean ± SD	15.37 ± 2.13	13.58 ± 3.38	14.76 ± 2.75	**0.005**^2^	**0.003**	0.682	0.294
AL (mm), mean ± SD	23.39 ± 0.97	23.23 ± 0.76	23.00 ± 0.94	0.179^2^			
Duration of the disease (years), median (Q1;Q3)	-	4.00 (2.00; 10.00)	5.00 (2.00; 10.00)	0.817^3^			
Pulmonary involvement, *n* (%)	-	18 (81.8)	4 (36.4)	**0.018**^1^			
Cardiac involvement, *n* (%)	-	8 (36.4)	3 (27.3)	0.709^1^			
Joint involvement, *n* (%)	-	8 (36.4)	8 (72.7)	0.071			
Digital ulcers (present/history), *n* (%)	-	9 (40.9)	2 (18.2)	0.259^1^			
Anti-Scl70 positive, *n* (%)	-	16 (80.0)	0 (0.0)	** < 0.001**^1^			
Anti-centromere positive, *n* (%)	-	2 (10.0)	5 (50.0)	**0.026**^1^			
Other Abs positive, *n* (%)	-	8 (40.0)	5 (50.0)	0.091^1^			

Abbreviations: *Abs* antibodies, *AL* axial length, *CRP* C-reactive protein, *dSSc* diffuse SSc, *ESR* erythrocyte sedimentation rate, *F* female, *h* hours, *IOP* intraocular pressure, *lSSc* limited SSc, *M* male, *MAP* mean arterial pressure, *NFC* nailfold capillaroscopy, *SSc* systemic sclerosis Notes: groups compared with chi-square test or Fisher exact test^1^ for nominal variables and with ANOVA^2^ or Kruskal–Wallis test^3^ for continuous variables. Post hoc tests used: Tukey test for ANOVA. *p* < 0.05 was considered statistically significant (highlighted with bold)

Choroidal parameters were compared between the SSc and control groups (Table 3). Lower choroidal thickness and volume values were demonstrated in various ETDRS subfields for the SSc group. The CVI was significantly higher in patients with SSc, whereas the TCA, LA, and SA were significantly lower in SSc patients than in the control group.

**Table 3:** Choroidal parameters comparison between eyes of SSc patients and control group

Variable	SSc mean ± SD	Control group mean ± SD	MD	95% CI	*p*	*p*_adj*_
Choroidal thickness (μm):						
Outer T	252.64 ± 45.91	267.86 ± 60.94	− 15.22	− 33.20; 2.77	0.097	0.114
Inner T	279.98 ± 60.20	301.82 ± 74.54	− 21.84	− 44.54; 0.86	0.059	0.084
Central Macular	286.63 ± 64.83	318.35 ± 82.20	− 31.73	− 56.49; − 6.97	**0.012**	**0.043**
Outer N	223.68 ± 64.12	241.81 ± 64.02	− 18.13	− 39.95; 3.69	0.103	0.114
Inner N	265.44 ± 66.35	297.86 ± 84.91	− 32.42	− 57.89; − 6.95	**0.013**	**0.043**
Outer S	286.46 ± 63.36	311.22 ± 67.28	− 24.76	− 46.91; − 2.61	**0.029**	0.058
Inner S	285.22 ± 65.25	323.58 ± 76.68	− 38.36	− 62.31; − 14.41	**0.002**	**0.020**
Outer I	255.51 ± 59.85	268.08 ± 74.02	− 12.57	− 35.12; 9.98	0.272	0.272
Inner I	270.36 ± 58.58	293.76 ± 80.93	− 23.40	− 46.89; 0.08	0.051	0.084
SFCT	288.92 ± 65.23	318.53 ± 88.80	− 29.62	− 55.55; − 3.69	**0.025**	0.058
Choroidal volume (mm^3^):						
Outer T	1.34 ± 0.25	1.42 ± 0.32	− 0.08	− 0.17; 0.02	0.122	0.136
Inner T	0.44 ± 0.09	0.47 ± 0.12	− 0.03	− 0.07; 0.002	0.068	0.097
Central macular	0.23 ± 0.05	0.25 ± 0.06	− 0.02	− 0.04; − 0.01	**0.013**	**0.035**
Outer N	1.17 ± 0.30	1.26 ± 0.35	− 0.09	− 0.20; 0.02	0.093	0.116
Inner N	0.42 ± 0.10	0.47 ± 0.13	− 0.05	− 0.09; − 0.01	**0.007**	**0.035**
Outer S	1.50 ± 0.36	1.66 ± 0.36	− 0.16	− 0.28; − 0.03	**0.014**	**0.035**
Inner S	0.45 ± 0.10	0.51 ± 0.12	− 0.06	− 0.10; − 0.02	**0.003**	**0.030**
Outer I	1.35 ± 0.32	1.40 ± 0.39	− 0.05	− 0.17; 0.07	0.419	0.419
Inner I	0.42 ± 0.09	0.46 ± 0.13	− 0.04	− 0.08; − 0.002	**0.041**	0.068
Total	7.35 ± 1.48	7.92 ± 1.76	− 0.57	− 1.12; − 0.02	**0.041**	0.068
Other choroidal parameters:						
TCA (μm^2^)	323 181.03 ± 64 155.42	360 476.63 ± 88 764.76	− 37,295.60	− 63,436.91; − 11,154.28	**0.006**	**0.012**
LA (μm^2^)	217 247.28 ± 42 660.47	238 322.08 ± 56 400.96	− 21,074.80	− 38,002.76; − 4146.84	**0.015**	**0.020**
SA (μm^2^)	105 933.75 ± 23 574.39	122 154.55 ± 34 367.12	− 16,220.80	− 26,136.48; − 6305.11	**0.002**	**0.008**
CVI (%)	67.26 ± 2.63	66.30 ± 2.82	0.97	0.03; 1.90	**0.043**	**0.043**

Abbreviations: *CI* confidence interval, *CVI* choroidal vascularity index, *I* inferior, *LA* luminal area, *MD* mean difference, *N* nasal, *SSc* systemic sclerosis, *S* superior, *SFCT* subfoveal choroidal thickness, *SA* stromal area, *T* temporal, *TCA* total choroidal area. Conventional ETDRS grid with nine subfields, central macular field (central field within a 500-µm radius), four inner subfields (within a 500–1500-µm radius) and four outer subfields (within a 1500–3000-µm radius) Notes: data presented as mean ± SD. Groups compared with *t*-test. *p* < 0.05 was considered statistically significant (highlighted with bold)

^*^p value after Benjamini–Hochberg correction for multiple comparisons. Correction was made separately for: choroidal thickness parameters (10 comparisons), choroidal volume parameters (10 comparisons), and other choroidal parameters (4 comparisons)

No significant differences in choroidal thickness and volume or other choroidal parameters were found within the SSc subtypes (Table 4) or between eyes stratified according to SSc pattern (early, active, or late) using nailfold capillaroscopy (*p* > 0.05 for all) (Table 5). For the comparison of choroidal parameters between groups, correction for multiple comparisons was applied (Tables 3, 4, and 5), without a significant impact on the overall results.

**Table 4:** Choroidal parameters comparison of SSc patients stratified according to the subtypes versus controls

Variable	Control group, mean ± SD	dSSc, mean ± SD	lSSc, mean ± SD	*p*	*p*_adj*_	Post hoc		
						Controls vs. dSSc	Controls vs. lSSc	dSSc vs. lSSc
Choroidal thickness (μm):								
Outer T	267.86 ± 60.94	254.79 ± 47.16	248.45 ± 44.24	0.257	0.286			
Inner T	301.82 ± 74.54	280.59 ± 56.45	278.80 ± 68.47	0.188	0.235			
Central Macular	318.35 ± 82.20	286.44 ± 58.79	287.00 ± 76.93	0.054	0.143			
Outer N	241.81 ± 64.02	231.62 ± 67.47	208.20 ± 55.36	0.109	0.182			
Inner N	297.86 ± 84.91	265.31 ± 64.21	265.70 ± 72.07	0.057	0.143			
Outer S	311.22 ± 67.28	294.08 ± 64.79	271.60 ± 59.23	**0.044**	0.143	0.377	**0.045**	0.427
Inner S	323.58 ± 76.68	288.72 ± 63.29	278.40 ± 70.07	**0.009**	0.090	**0.039**	**0.036**	0.862
Outer I	268.08 ± 74.02	257.23 ± 64.42	252.15 ± 51.16	0.548	0.548			
Inner I	293.76 ± 80.93	271.74 ± 57.50	267.65 ± 62.08	0.172	0.235			
SFCT	318.53 ± 88.80	286.64 ± 60.83	293.35 ± 74.54	0.098	0.182			
Choroidal volume (mm^3^):								
Outer T	1.42 ± 0.32	1.36 ± 0.26	1.30 ± 0.24	0.257	0.286			
Inner T	0.47 ± 0.12	0.44 ± 0.09	0.44 ± 0.11	0.205	0.256			
Central macular	0.25 ± 0.06	0.23 ± 0.05	0.22 ± 0.06	0.055	0.138			
Outer N	1.26 ± 0.35	1.20 ± 0.31	1.12 ± 0.28	0.184	0.256			
Inner N	0.47 ± 0.13	0.42 ± 0.10	0.41 ± 0.11	**0.031**	0.103	0.077	0.107	0.951
Outer S	1.66 ± 0.36	1.53 ± 0.38	1.44 ± 0.31	**0.031**	0.103	0.187	**0.047**	0.622
Inner S	0.51 ± 0.12	0.46 ± 0.10	0.44 ± 0.11	**0.011**	0.103	0.058	**0.035**	0.799
Outer I	1.40 ± 0.39	1.36 ± 0.34	1.34 ± 0.27	0.701	0.701			
Inner I	0.46 ± 0.13	0.43 ± 0.09	0.42 ± 0.10	0.146	0.243			
Total	7.92 ± 1.76	7.45 ± 1.50	7.14 ± 1.46	0.109	0.218			
Other choroidal parameters:								
TCA (μm^2^)	360 476.63 ± 88 764.76	315 500.56 ± 59 521.34	338 946.18 ± 71 863.48	**0.017**	**0.034**	**0.013**	0.539	0.540
LA (μm^2^)	238 322.08 ± 56 400.96	213 195.58 ± 41 928.53	225 563.92 ± 44 081.19	**0.045**	0.060	**0.036**	0.593	0.662
SA (μm^2^)	122 154.55 ± 34 367.12	102 304.99 ± 19 824.10	113 382.26 ± 29 036.73	**0.004**	**0.016**	**0.003**	0.494	0.389
CVI (%)	66.30 ± 2.82	67.45 ± 2.60	66.88 ± 2.73	0.102	0.102			

Abbreviations: *CVI* choroidal vascularity index, *dSSc* diffuse SSc, *I* inferior, *lSSc* limited SSc, *LA* luminal area, *N* nasal, *SSc* systemic sclerosis, *S* superior, *SFCT* subfoveal choroidal thickness, *SA* stromal area, *T* temporal, *TCA* total choroidal area. Conventional ETDRS grid with nine subfields, central macular field (central field within a 500-µm radius), four inner subfields (within a 500–1500-µm radius), and four outer subfields (within a 1500–3000-µm radius) Notes: data presented as mean ± SD. Groups compared with ANOVA. Post hoc tests used: Tukey test for ANOVA. *p* < 0.05 was considered statistically significant (highlighted with bold). *p value after Benjamini–Hochberg correction for multiple comparisons. Correction was made separately for: choroidal thickness parameters (10 comparisons), choroidal volume parameters (10 comparisons), and other choroidal parameters (4 comparisons)

**Table 5:** Comparison of choroidal parameters between eyes stratified according to SSc pattern on nailfold capillaroscopy

	Early SSc pattern	Active SSc pattern	Late SSc pattern	*p*	*p*_adj*_
Choroidal thickness (μm):					
Outer T	258.29 ± 56.18	249.14 ± 40.66	252.79 ± 44.90	0.816	0.966
Inner T	282.41 ± 70.40	275.71 ± 57.07	285.57 ± 56.77	0.869	0.966
Central Macular	289.59 ± 74.70	281.86 ± 65.00	292.57 ± 54.89	0.863	0.966
Outer N	235.76 ± 84.83	208.25 ± 56.20	239.86 ± 44.04	0.213	0.966
Inner N	262.12 ± 70.55	259.96 ± 65.44	280.43 ± 65.59	0.630	0.966
Outer S	288.82 ± 72.88	279.43 ± 66.55	297.64 ± 43.91	0.676	0.966
Inner S	288.00 ± 73.52	277.39 ± 66.49	297.50 ± 53.43	0.636	0.966
Outer I	249.82 ± 64.36	252.39 ± 53.42	268.64 ± 68.61	0.644	0.966
Inner I	268.24 ± 63.65	271.14 ± 56.79	271.36 ± 60.16	0.985	0.985
SFCT	299.12 ± 76.13	280.89 ± 64.10	292.57 ± 55.02	0.651	0.966
Choroidal Volume (mm3)					
Outer T	1.36 ± 0.29	1.31 ± 0.23	1.38 ± 0.25	0.630	0.926
Inner T	0.45 ± 0.11	0.44 ± 0.09	0.45 ± 0.09	0.904	0.995
Central macular	0.23 ± 0.06	0.22 ± 0.05	0.23 ± 0.04	0.847	0.995
Outer N	1.19 ± 0.36	1.12 ± 0.30	1.25 ± 0.24	0.435	0.926
Inner N	0.43 ± 0.12	0.41 ± 0.10	0.44 ± 0.08	0.642	0.926
Outer S	1.53 ± 0.39	1.45 ± 0.40	1.57 ± 0.24	0.536	0.926
Inner S	0.46 ± 0.11	0.44 ± 0,10	0.47 ± 0.09	0.610	0.926
Outer I	1.33 ± 0.34	1.34 ± 0.28	1.42 ± 0.36	0.648	0.926
Inner I	0.42 ± 0.10	0.42 ± 0.09	0.43 ± 0.10	0.995	0.995
Total	7.39 ± 1.78	7.18 ± 1.40	7.63 ± 1.29	0.641	0.926
Other choroidal parameters:					
TCA (μm^2^)	331,813.70 ± 69,467.72	316,810.48 ± 67,641.16	327,921.50 ± 51,425.26	0.734	0.889
LA (μm^2^)	223,529.93 ± 47,807.53	212,188.40 ± 45,001.79	221,672.38 ± 30,812.96	0.650	0.889
SA (μm^2^)	108,283.77 ± 23,328.90	104,622.08 ± 24,232.33	106,249.12 ± 23,968.39	0.889	0.889
CVI (%)	67.26 ± 2.39	67.01 ± 2.52	67.85 ± 3.22	0.639	0.889

Abbreviations: *SSc* systemic sclerosis, *T* temporal, *I* inferior, *N* nasal, *S* superior, *SFCT* subfoveal choroidal thickness, *TCA* total choroidal area, *LA* luminal area, *SA* stromal area, *CVI* choroidal vascularity index;; conventional ETDRS grid with nine subfields, central macular field (central field within a 500-µm radius), four inner subfields (within a 500–1500-µm radius), and four outer subfields (within a 1500–3000-µm radius) Notes: data presented as mean ± SD, unless otherwise indicated. Groups compared with ANOVA or Kruskal–Wallis test. Post hoc tests used: Tukey test for ANOVA, Dunn test for Kruskal–Wallis test. *p* < 0.05 was considered statistically significant. *p value after Benjamini–Hochberg correction for multiple comparisons. Correction was made separately for: choroidal thickness parameters (10 comparisons), choroidal volume parameters (10 comparisons), and other choroidal parameters (4 comparisons)

The univariate regression analyses of the associations between the choroidal parameters (CVI and central macular choroidal thickness and volume) and clinical, demographic, and ocular features are presented in Table 6 (SSc group) and Table 7 (control group). No significant associations were found between the choroidal parameters and age, sex, AL, nicotine use, MAP, duration of the disease, SSc subtype, scleroderma pattern in NFC, antibody profile, organ involvement, or medications that could affect choroidal parameters in the SSc group (except for Ca-blocker use and CVI). No associations of clinical and choroidal parameters were found in the control subjects.

**Table 6:** Regression analysis testing factors associated with choroidal parameters in SSc group

	^CVI^						^Choroidal thickness central macular^						^Choroidal volume central macular^					
	^*β*^	^SE^	^*B*^	^*p*^	^*R*2^	^Pseudo*R*2^	^*β*^	^SE^	^*B*^	^*p*^	^*R*2^	^Pseudo*R*2^	^*β*^	^SE^	^*B*^	^*p*^	^*R*2^	^Pseudo*R*2^
^Age, years^	− 0.046	0.039	− 0.220	0.241	0.049	0.015	− 1.301	1.064	− 0.236	0.232	0.052	0.017	− 0.001	0.001	− 0.378	0.053	0.132	0.099
^Sex. male^	0.756	1.127	−	0.508	0.016	− 0.019	3.679	28.572	-	0.899	0.001	− 0.036	0.008	0.019	-	0.695	0.006	− 0.031
^Nicotine^	0.922	1.917	−	0.634	0.008	− 0.027	23.140	41.710	-	0.584	0.011	− 0.025	0.026	0.029	-	0.378	0.029	0.026
^AL^	− 0.548	0.586	− 0.172	0.358	0.030	− 0.004	− 25.090	17.930	− 0.302	0.173	0.068	0.033	− 0.009	0.013	− 0.155	0.483	0.018	− 0.018
^MAP^	0.019	0.054	0.068	0.729	0.005	− 0.032	− 1.067	1.439	− 0.146	0.465	0.022	− 0.017	0.000	0.001	0.033	0.874	0.001	− 0.039
^CVI^	-	-	-	-	-	-	− 3.260	4.634	− 0.125	0.488	0.019	− 0.019	− 0.002	0.003	− 0.081	0.645	0.009	− 0.031
^TCA^	0.000	0.000	− 0.140	0.460	0.019	− 0.015	0.001	0.000	0.807	** < 0.001**	0.767	0.758	0.000	0.000	0.806	** < 0.001**	0.698	0.686
^LA^	0.000	0.000	0.045	0.813	0.002	− 0.034	0.001	0.000	0.782	** < 0.001**	0.728	0.717	0.000	0.000	0.775	** < 0.001**	0.666	0.652
^SA^	0.000	0.000	− 0.456	**0.011**	0.208	0.179	0.002	0.000	0.770	** < 0.001**	0.692	0.679	0.000	0.000	0.757	** < 0.001**	0.605	0.589
^Choroidal thickness central macular^	− 0.006	0.009	− 0.156	0.488	0.019	− 0.019	-	-	-	-	-	-	0.001	0.000	1.120	** < 0.001**	0.992	0.992
^Choroidal volume total^	− 0.204	0.357	− 0.123	0.574	0.012	− 0.026	39.102	3.226	0.905	** < 0.001**	0.845	0.839	0.029	0.002	0.955	** < 0.001**	0.873	0.868
^dSSc/lSSc, ISSc^	− 0.811	1.007	-	0.427	0.023	− 0.012	− 8.526	26.825	-	0.753	0.004	− 0.033	− 0.021	0.019	-	0.261	0.047	0.011
^NFC pattern. active=baseline^																		
^Early^	0.537	1.165	-	0.649	0.014	− 0.059	− 0.240	31.344	-	0.994	0.009	− 0.066	0.011	0.021	-	0.613	0.036	− 0.038
^Late^	0.652	1.218	-	0.597			14.635	31.344	-	0.644			0.021	0.021	-	0.337		
^Duration of the disease^	− 0.083	0.068	− 0.237	0.235	0.050	0.016	2.449	1.799	0.268	0.185	0.064	0.029	0.001	0.001	0.092	0.683	0.006	− 0.031
^Anti−Scl70 positive^	0.598	1.048	-	0.574	0.013	− 0.027	51.380	34.840	-	0.153	0.080	0.043	0.022	0.020	-	0.288	0.045	0.007
^Ant−centromere positive^	0.207	1.202	-	0.865	0.001	− 0.039	− 57.640	42.110	-	0.183	0.069	0.033	− 0.033	0.024	-	0.186	0.069	0.032
^Joint involvement^	− 1.091	0.940	-	0.256	0.046	0.012	16.880	25.360	-	0.511	0.016	− 0.020	0.004	0.018	-	0.846	0.001	− 0.036
^Pulmonary involvement^	0.983	0.979	-	0.324	0.035	0.000	− 14.830	26.720	-	0.584	0.011	− 0.025	− 0.016	0.018	-	0.383	0.028	− 0.008
^Cardiac involvement^	− 1.751	0.994	-	0.089	0.099	0.068	− 8.832	26.821	-	0.744	0.004	− 0.033	− 0.019	0.019	-	0.299	0.039	0.004
^Digital ulcers^	0.484	1.044	-	0.646	0.008	− 0.028	25.970	26.410	-	0.334	0.035	− 0.001	0.028	0.018	-	0.121	0.087	0.053
^PDE inhibitors^	0.274	0.961	-	0.778	0.003	− 0.033	0.995	25.562	-	0.969	0.000	− 0.037	0.012	0.018	-	0.510	0.016	− 0.020
^Ca−blocker^	2.109	1.010	-	**0.046**	0.135	0.104	− 16.640	27.420	-	0.549	0.013	− 0.023	− 0.004	0.019	-	0.847	0.001	− 0.036
^Hydroxychoroquine^	0.105	1.135	-	0.927	0.000	− 0.035	− 7.364	29.818	-	0.807	0.002	− 0.035	− 0.028	0.021	-	0.201	0.059	0.025
^Steroids^	1.759	0.910	-	0.064	0.118	0.086	− 4.966	25.668	-	0.848	0.001	− 0.036	− 0.016	0.018	-	0.370	0.029	− 0.006
^Diuretic^	0.009	1.048	-	0.993	0.000	− 0.036	7.653	26.834	-	0.778	0.003	− 0.034	− 0.009	0.019	-	0.657	0.007	− 0.029

Abbreviations: *AL* axial length, *CVI* choroidal vascularity index, *dSSc* diffuse SSc, *lSSc* limited SSc, *LA* luminal area, *MAP* mean arterial pressure, *NFC* nailfold capillaroscopy, *PDE* phosphodiesterase, *SA* stromal area, *SSc* systemic sclerosis, *TCA* total choroidal area, *β* beta coefficient, *B* standardized beta

Only one eye per patient included into the analysis, *p* < 0.05 highlighted with bold

**Table 7:** Regression analysis testing factors associated with choroidal parameters in control group

	^CVI^						^Choroidal thickness central macular^						^Choroidal volume central macular^					
	^*β*^	^SE^	^*B*^	^*p*^	^*R*2^	^Pseudo*R*2^	^*β*^	^SE^	^*B*^	^*p*^	^*R*2^	^Pseudo*R*2^	^*β*^	^SE^	^*B*^	^*p*^	^*R*2^	^Pseudo*R*2^
^Age, years^	0.005	0.047	0.019	0.912	0.000	− 0.027	− 1.782	1.292	− 0.220	0.176	0.049	0.023	− 0.001	0.001	− 0.226	0.164	0.052	0.026
^Sex. male^	− 0.667	0.974	-	0.498	0.013	− 0.015	8.024	27.821	-	0.775	0.002	− 0.025	0.009	0.022	-	0.671	0.005	− 0.022
^Nicotine^	− 1.643	1.299	-	0.214	0.043	0.016	48.640	37.440	-	0.202	0.044	0.018	0.036	0.029	-	0.226	0.039	0.013
^AL^	− 0.549	0.504	− 0.181	0.284	0.032	0.005	− 11.510	14.690	− 0.131	0.438	0.016	− 0.010	− 0.008	0.012	− 0.121	0.474	0.014	− 0.013
^MAP^	− 0.022	0.044	− 0.093	0.620	0.009	− 0.026	− 0.568	1.756	− 0.066	0.749	0.004	− 0.032	0.000	0.001	− 0.043	0.833	0.002	− 0.034
^CVI^	-	-	-	-	-	-	− 8.265	4.605	− 0.286	0.081	0.082	0.057	− 0.007	0.004	− 0.292	0.075	0.085	0.059
^TCA^	0.000	0.000	− 0.235	0.155	0.055	0.029	0.001	0.000	0.821	** < 0.001**	0.678	0.669	0.000	0.000	0.822	** < 0.001**	0.674	0.665
^LA^	0.000	0.000	− 0.061	0.716	0.004	− 0.024	0.001	0.000	0.794	** < 0.001**	0.634	0.623	0.000	0.000	0.792	** < 0.001**	0.627	0.616
^SA^	0.000	0.000	0.505	**0.001**	0.255	0.234	0.002	0.000	0.808	** < 0.001**	0.656	0.647	0.000	0.000	0.811	** < 0.001**	0.657	0.647
^Choroidal thickness central macular^	− 0.010	0.006	− 0.287	0.081	0.082	0.057	-	-	-	-	-	-	0.001	0.000	0.998	** < 0.001**	0.995	0.995
^Choroidal volume total^	− 0.539	0.301	− 0.293	0.082	0.084	0.058	47.090	3.556	0.887	** < 0.001**	0.829	0.825	0.037	0.003	0.877	** < 0.001**	0.814	0.809

Abbreviations: *AL* axial length, *CVI* choroidal vascularity index, *LA* luminal area, *MAP* mean arterial pressure, *SA* stromal area, *TCA* total choroidal area, *β* beta coefficient*, B* standardized beta

Only one eye per patient included into the analysis, *p* < 0.05 highlighted with bold

The CVI was calculated as LA/TCA, and the TCA consisted of the LA and SA. Consequently, associations among these parameters were detected in univariate regression analyses, as well as between choroidal thickness and volume.

## Discussion

Detailed studies on choroidal thickness have found that patients with SSc have significantly thinner macular choroids than healthy subjects [7–9, 17–20], which is consistent with our results. However, these studies have been based on measurements taken at various points and at different distances from the fovea. To the best of our knowledge, the present study was the first to analyze choroidal thickness and volume over a 6-mm diameter in the macula in SSc patients. The mean choroidal thickness and volume were obtained for each ETDRS subfield. According to Singh et al., even an accurate estimate of choroidal thickness from a few sampling points could be inadequate for assessing choroidal involvement due to irregularities in the choroidal–scleral junction. Hence, a volumetric analysis of the choroid is preferable [26]. Hirata et al. confirmed the asymmetric nature of macular choroidal thickness in healthy subjects, with a significantly thinner inferior choroid than superior choroid, and the outer nasal choroid was significantly thinner than all other areas in the ETDRS ring [35], which was also reflected in our results in both the control and SSc groups. We confirmed significantly lower subfoveal choroidal thickness (SFCT) values and thinner choroids within various ETDRS subfields for patients with SSc compared to the control group and, consequently, lower choroidal volume with a significantly lower total volume of the choroid. In addition to choroidal thickness, a few studies have focused on the specific choroidal vessel layer thicknesses. Using EDI-OCT scans, Ranjbar et al. manually measured subfoveal thicknesses in SSc patients and revealed thinner Sattler’s and Haller’s layers in contrast to unchanged thickness of the choriocapillaris layer. They speculated that the choriocapillaris remained morphologically unchanged in connection with proximity to the retinal pigment epithelium, which is a major source of vascular endothelial growth factor A, enhancing endothelial cell survival. Simultaneously, submacular perfusion (determined by the binarization of OCT-A angiograms) was significantly reduced in all three vascular layers in patients with SSc compared to control patients [10]. Similar results were reported by Rommel et al., except for choriocapillaris perfusion, which did not differ significantly [8]. Additionally, Hekimsoy et al. reported no significant difference in choriocapillaris flow area in OCT-A [7].

In addition, in our study, we investigated not only the choroidal thickness but also the CVI. A CVI-measured area of 1000 μm in width centered on the fovea was determined to match the choroidal thickness and volume values from the central macular ETDRS ring of 1000-μm diameter. Our results showed that the CVI was significantly higher in patients with SSc than in healthy control subjects. Choroidal thickness is mainly determined by the thickness of the Sattler’s and Haller’s layers [8], and it was previously believed that the thinner choroid in patients with SSc was mainly due to vascular damage [18, 19], which was not reflected in the CVI in our study. The TCA, LA, and SA were significantly lower in SSc patients compared to control group. However, the significant difference in the stromal component was more pronounced than that in the luminal. This may suggest that both the luminal and stromal components were thinner, with the latter being more affected. Histopathological studies have shown endothelial cell damage, basement membrane thickening, the absence of pericytes, and the deposition of abnormal material in and around the endothelium in choroidal vessels in SSc patients [36]. The fibrotic process in SSc is characterized by the progressive tissue accumulation of extracellular matrix (ECM) protein-like collagens, elastin, glycosaminoglycans, tenascin, and fibronectin isoforms in the skin and multiple organs [1]. The question remains open whether luminal changes precede, accompany, or follow stromal changes. Carnevali et al. investigated abnormalities in retinal and choroidal vascular plexuses in patients with SSc using SD-OCT and OCT-A. In contrast to our results, they did not find a significant difference in the CVIs between the SSc and control groups. They revealed a significantly higher choriocapillaris plexus flow index, which was not within the scope of our study. They speculated that compensatory mechanisms occurred in order to counterbalance the increased oxygen demand of the retinal layers. Moreover, they suggested that the unaltered CVI, together with an increased choriocapillaris flow index, suggests that the choroidal vasculature does not seem to be primarily affected in SSc and hypothesized that increased choroidal vessel area is accompanied by a corresponding increase in the stromal compartment [37]. Unfortunately, they did not show data regarding choroidal thickness and the size of area used for CVI calculation, which makes comparison more difficult.

Structural microvascular abnormalities related to the pathophysiological process of SSc can be visualized noninvasively using the nailfold capillaroscopy technique [28]. The presence of giant capillaries is characteristic for “early” and “active” scleroderma patterns, while the presence of severe lowered density combined with abnormal shape is typical for the “late” scleroderma pattern [28]. A “late” SSc pattern represents the clearest aspect of advanced SSc microvascular damage, regardless of the presence of a limited or diffuse subtype [13]. Carnevali et al. performed a qualitative assessment of retinal microcirculation abnormalities, i.e., the presence of megacapillaries in the deep capillary plexus of the macular region, and 75% of SSc eyes presented with abnormalities [37]. Currently, there are no data available on the direct assessment of the choriocapillary layer for the presence of megacapillaries, although the increased CVIs in our study could have been partly caused by an increase in the diameter of the vascular component in the choroid or their tortuosity. Nevertheless, no significant differences in choroidal thickness or volume and other choroidal parameters were found between eyes stratified according to SSc pattern (“early,” “active,” or “late”) through NFC in our study.

We are well aware of the potential limitations of this study. The study group consisted of a relatively small number of patients, owing to the rarity of the disease. Moreover, the study was conducted during the COVID-19 pandemic, and the study period had to be shortened because scheduled hospital admissions were reduced. A longitudinal study could help to evaluate the potential usefulness of choroidal assessment for monitoring and prognosticating patients with SSc. Additionally, simultaneous assessment of the choroid using both SD-OCT and OCT-A, not only SD-OCT, would give a wider perspective. In future studies, improvements in CVI assessment and a fully automated CVI algorithm integrated into an OCT device may facilitate the standardization of this informative parameter, as pointed out by Agrawal et al. [38]. We were able to demonstrate altered choroidal parameters in patients with SSc, but the regression analyses did not identify clinical factors definitely associated with the CVI or central macular thickness and volume. This issue requires further study.

In conclusion, we were able to show thinning of the choroid in SSc patients. Our results shed new light on SSc pathogenesis in the eyes. Stromal involvement seemed to predominate in the vascular component. Consequently, fibrosis might outweigh microangiopathy. The CVI could serve as a diagnostic tool to determine the involvement of both choroidal components. These observations may direct future research.

### Supplementary Information

Below is the link to the electronic supplementary material.

Supplementary file1 (PDF 2517 KB)

## Data Availability

All materials and information are available upon an e-mail request to the corresponding authors. Names and exact data of the participants of the study may not be available because of privacy policies.
