# Supplementary material for: Macular choroidal thickness, volume, and vascularity index in patients with systemic sclerosis
Source: Graefes Arch Clin Exp Ophthalmol. 2023 Dec 22;262(5):1475–87. doi: 10.1007/s00417-023-06342-4 (PMC11031445; doi:10.1007/s00417-023-06342-4)
Supplement: Supplementary file 1 — Supplementary file1 (PDF 2517 KB) [file 417_2023_6342_MOESM1_ESM.pdf]

STEP-BY-STEP binarization of images and choroidal vascularity index (CVI) calculations

1. The 1 × 1 pixel image of the OCT single horizontal line scan of the macular region (30°) centered on the fovea (Heidelberg Engineering, Heidelberg, Germany) was opened in ImageJ software (https://imagej.nih.gov/ij, version 1.53k, U.S. National Institutes of Health, Bethesda, MD, USA).

2. The scale was set as follows; the image was enlarged and the line tool was used to measure a pixel length of 200 µm, as given in the horizontal scale at the bottom of the OCT scan.
   The known horizontal distance (yellow frame) was entered into the scale-set window. The horizontal scale was automatically calculated (red frame).

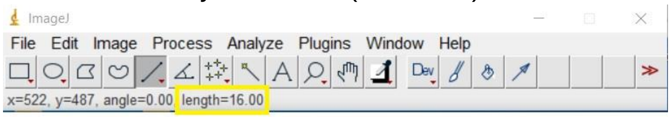

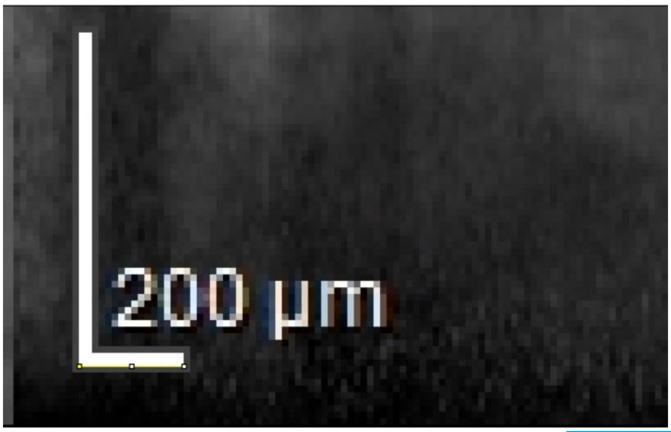

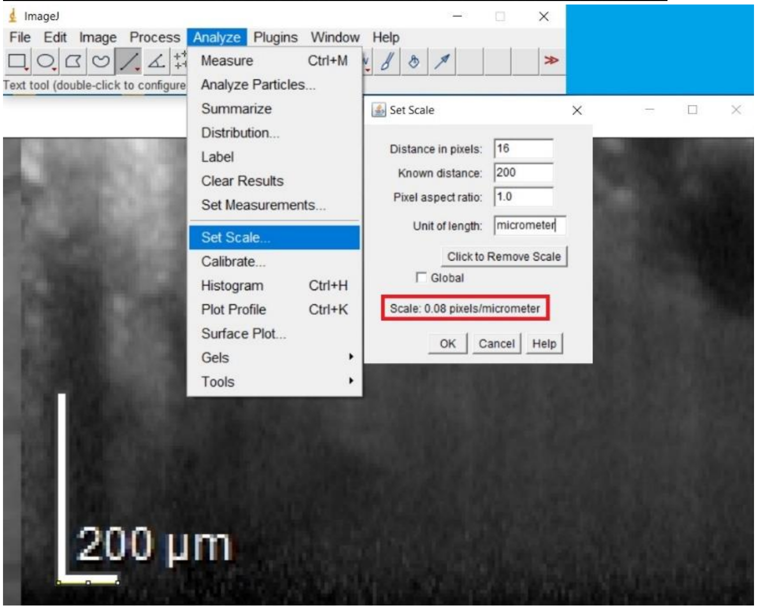

The scale was reset (green frame) and the vertical scale was calculated similarly.

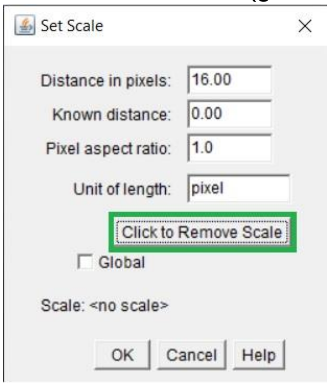

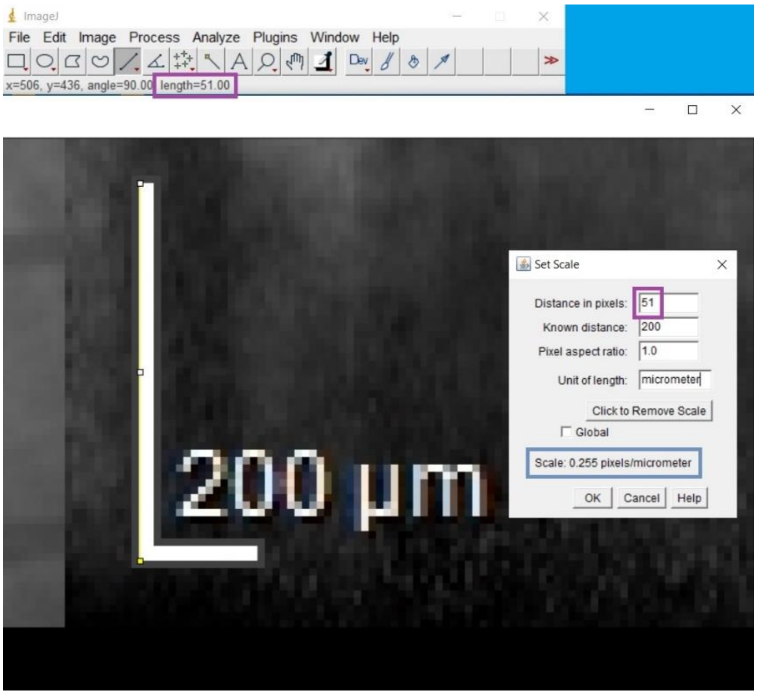

The pixel aspect ratio was calculated by dividing the horizontal pixel values by the vertical pixel values (16/51=0.31372549).
Once again, the horizontal scale was entered and the rounded result of the pixel aspect ratio (0.314) was entered into the pixel aspect ratio window. The unit of length was micrometers.

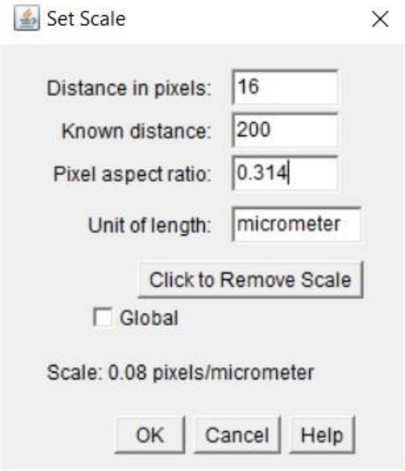

3. A scale bar of 1000 µm was entered into the picture.

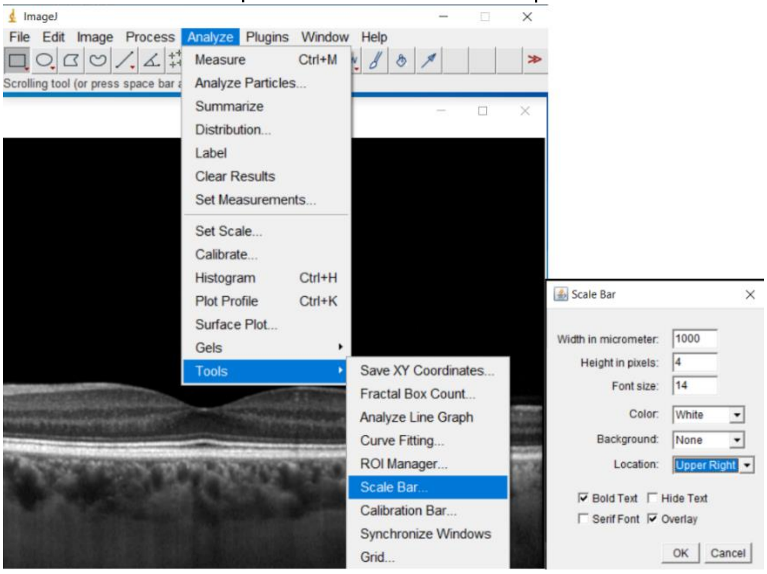

4. A line with a length of 1000 μm was marked with rectangular tool.

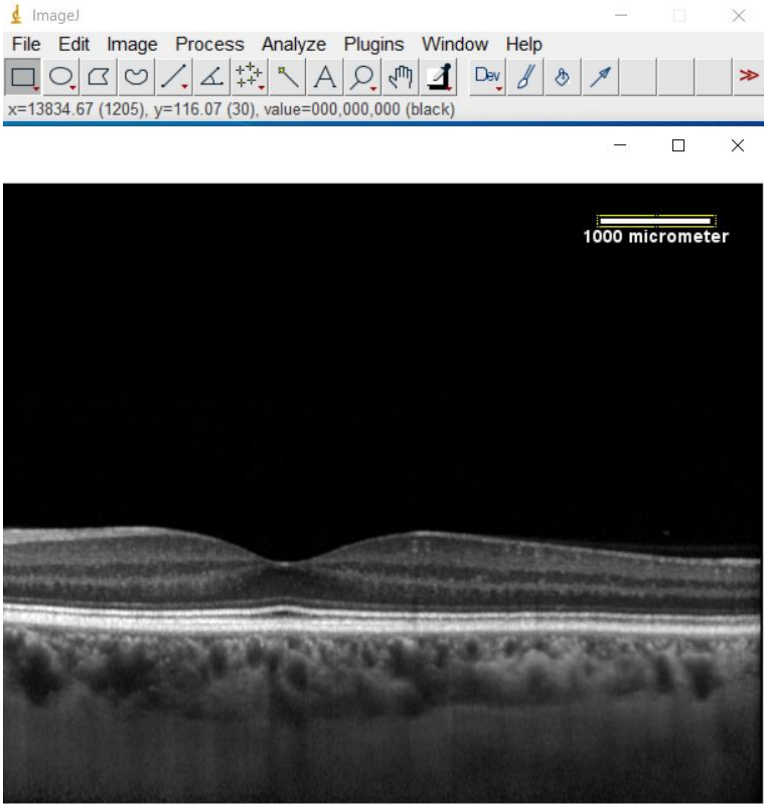

5. The line was moved above the center of the fovea by double-clicking the right mouse button on the line and then holding it down to easily delimit the area of interest.

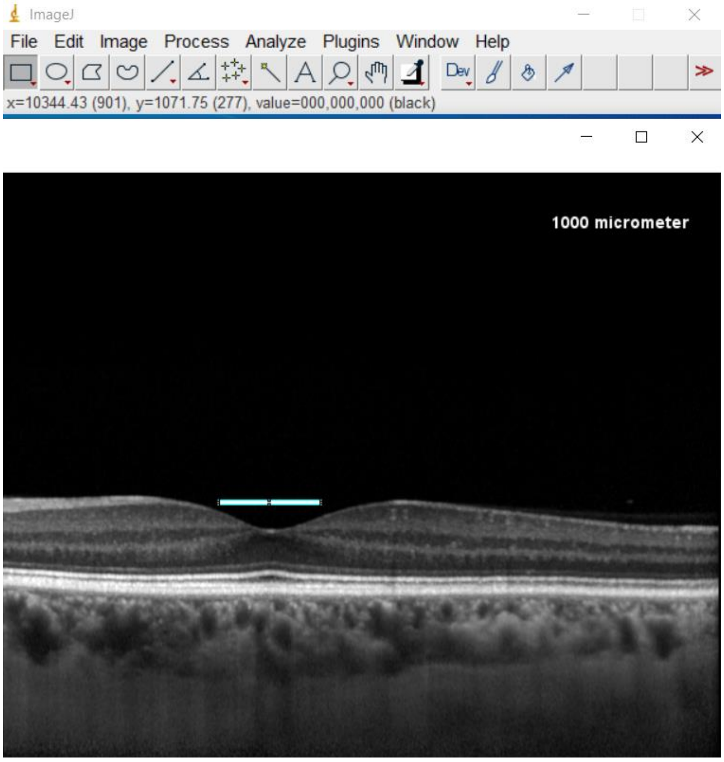

6. The polygon tool was used to select the area between the outer boundary of the RPE-Bruch's membrane layer and the choroidal-scleral junction with a length of 1000 μm.

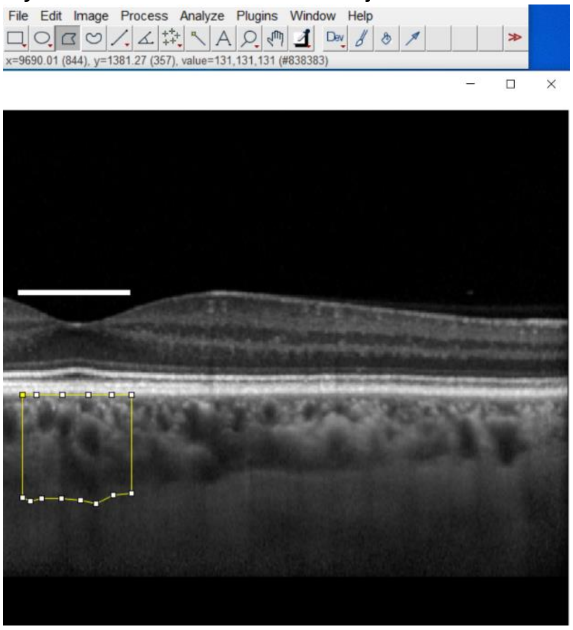

7. The polygon area representing the region of interest (ROI) was added to the ROI manager (by clicking the left mouse button on this area).

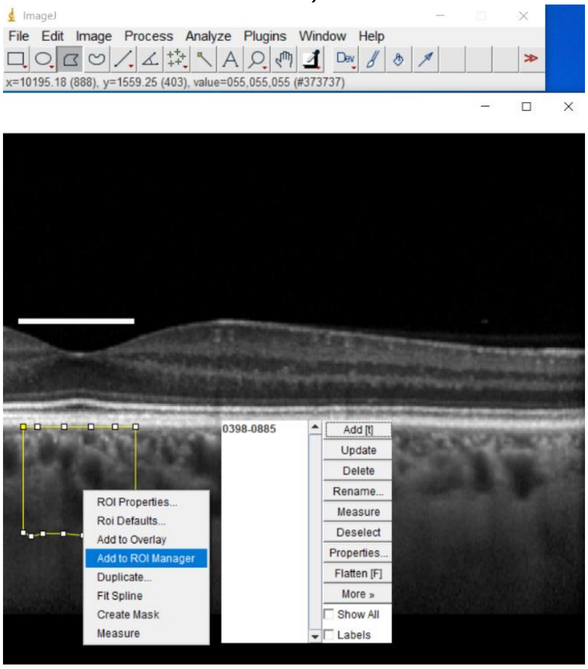

8. The image was converted to an 8-bit image.

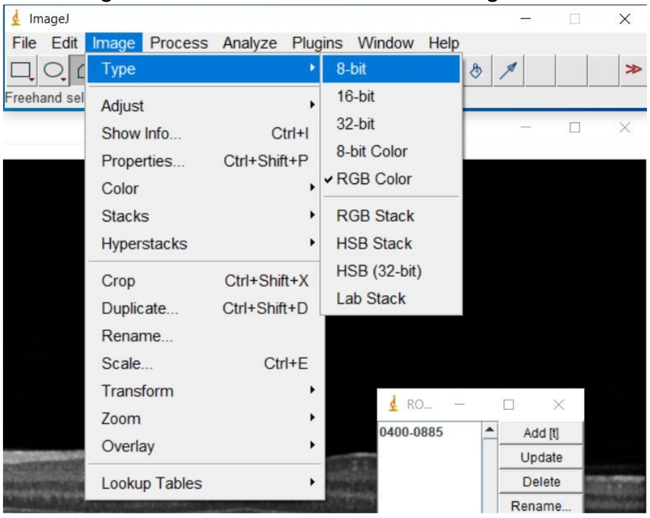

9. The image was adjusted to Niblack with the Auto Local Treshold Tool.

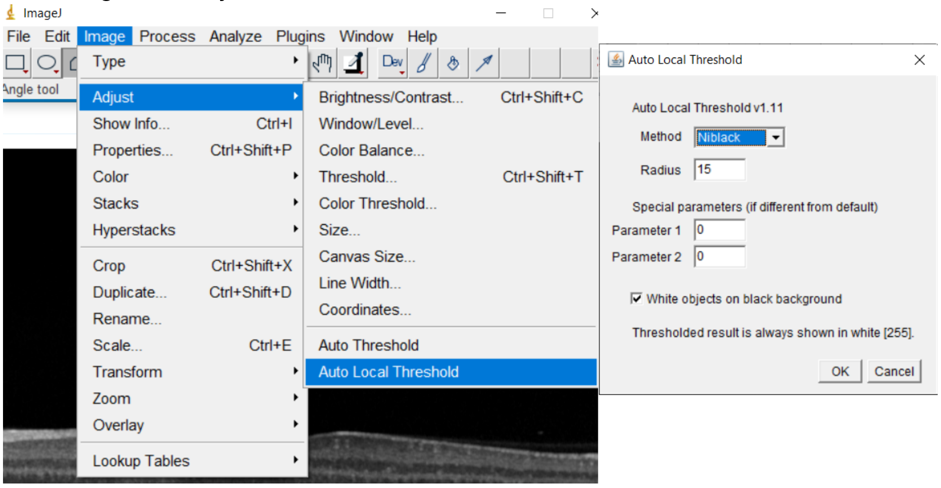

10. The binarized image was re-converted to an RGB image.

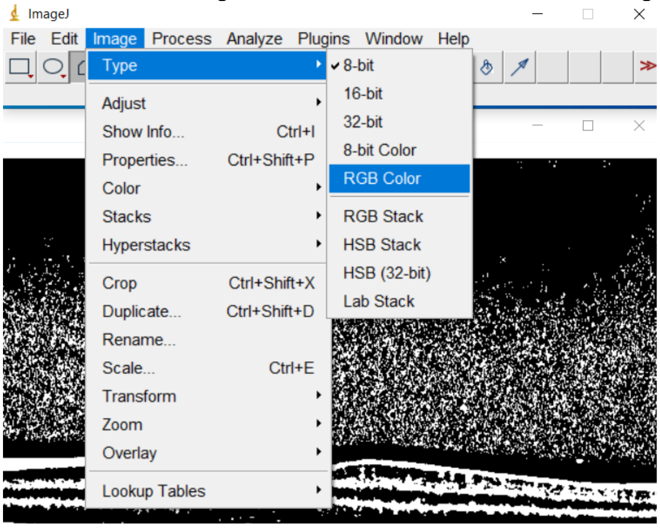

11. The area of vascularity was highlighted by selecting Color Threshold tool: the first bar under brightness was adjusted to 0, the second to 254 (blue frame) and confirmed by clicking SELECT.

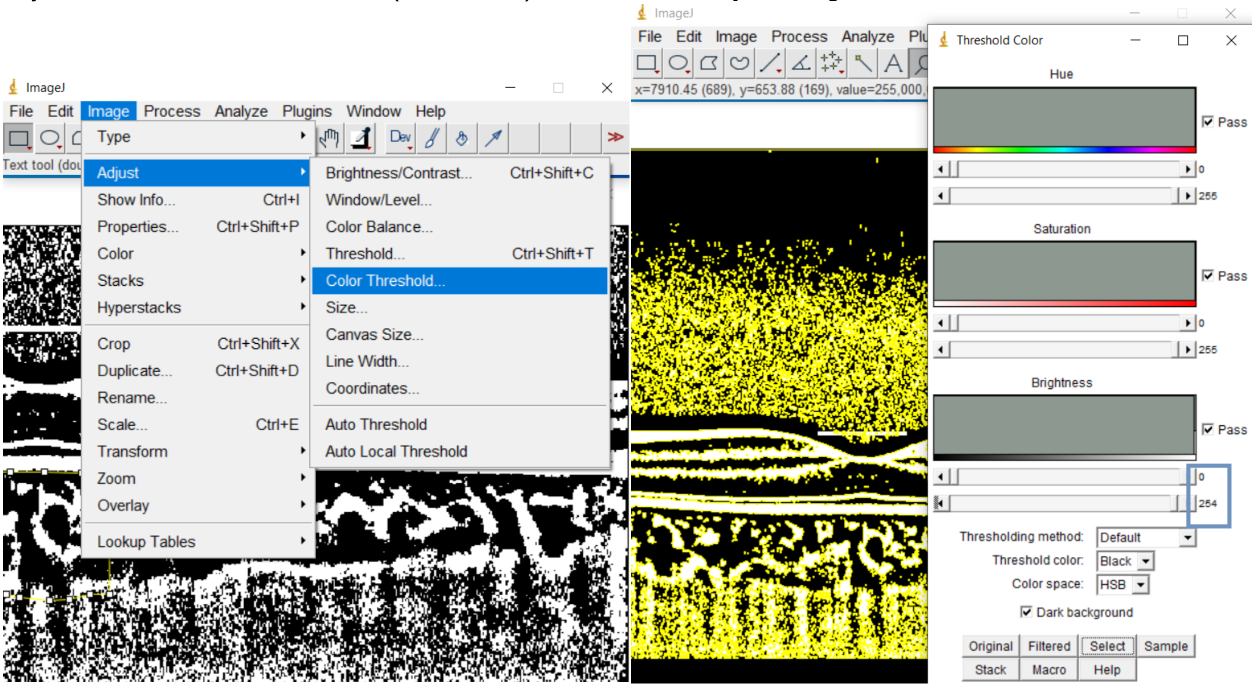

12. The highlighted area was added to the ROI manager (by clicking the left mouse button on this area).

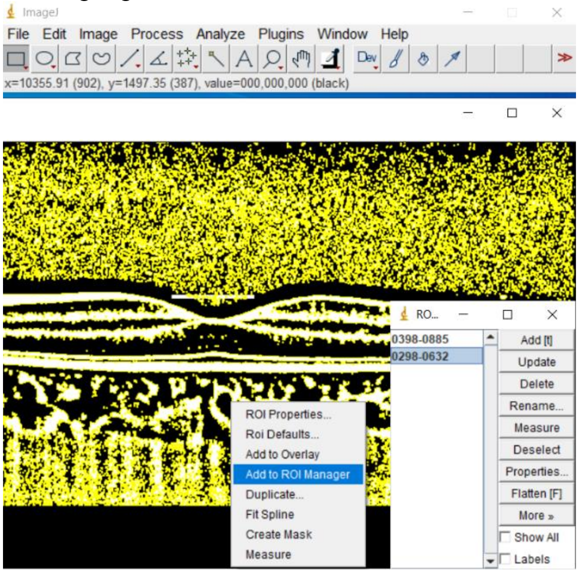

13. Both areas in the ROI manager were selected by holding the CTRL button on the keyboard and merging by selecting More < AND.

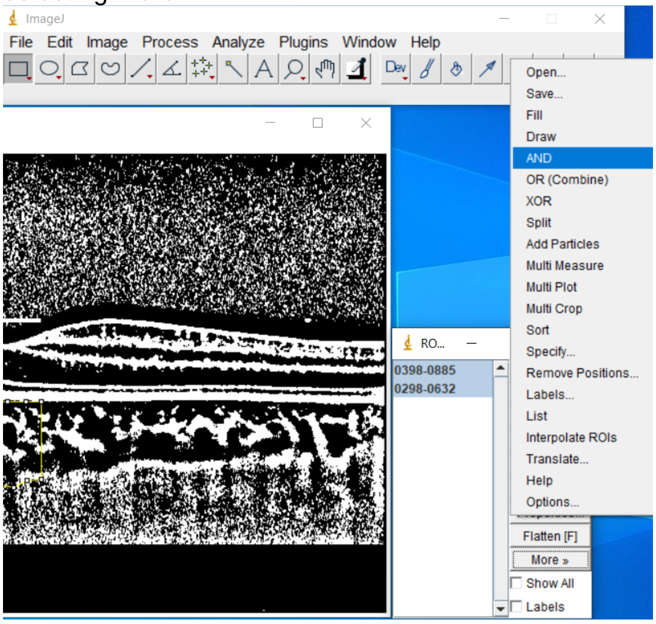

14. The highlighted area within the selected polygon was added to the ROI manager (by clicking the left mouse button in this area).

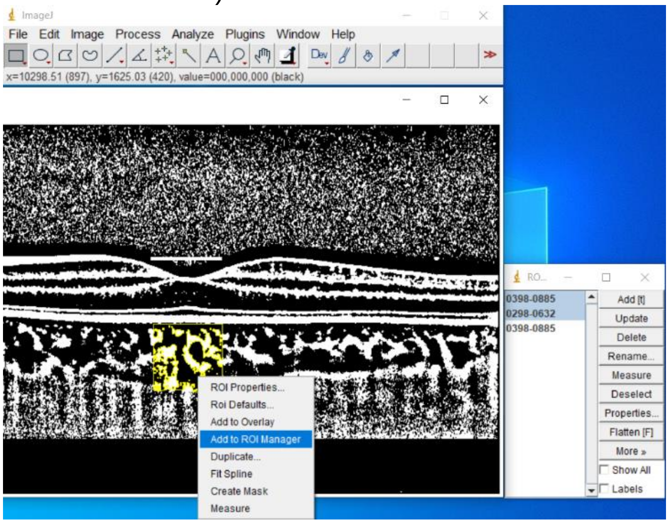

15. Lastly, the first area that corresponds to the total choroidal area (TCA) and the third area, which represents the luminal area (LA), were measured in the ROI manager by clicking the button MEASURE. The measured areas are displayed in square micrometers.

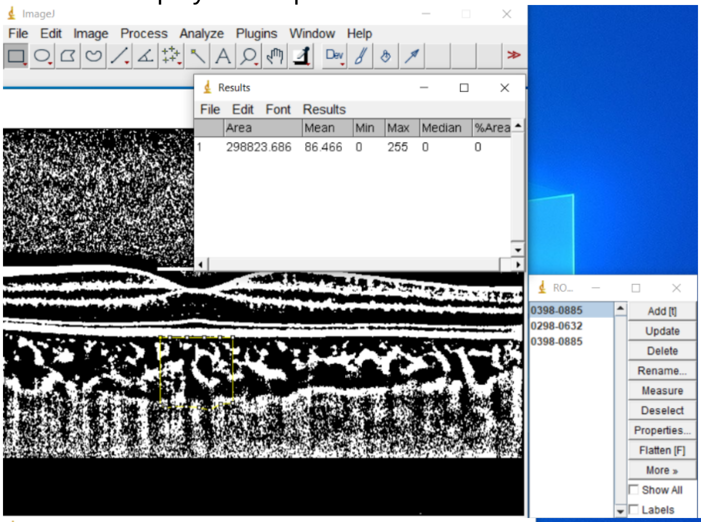

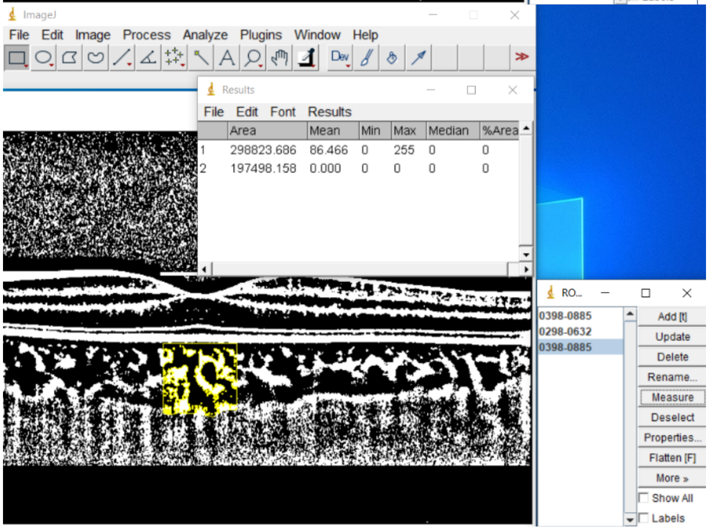

16. The stromal area (SA) was obtained after subtraction of the LA from the TCA: 298824-197498=101326 μm2

17. The choroidal vascularity index (CVI) was calculated: (197498/298824)x100%=66.09%
